# Spatio–temporal variation in stable isotope signatures (δ^13^C and δ^15^N) of sponges on the Saba Bank

**DOI:** 10.7717/peerj.5460

**Published:** 2018-08-14

**Authors:** Fleur C. Van Duyl, Benjamin Mueller, Erik H. Meesters

**Affiliations:** 1 Department of Marine Microbiology and Biogeochemistry, NIOZ Royal Netherlands Institute for Sea Research and Utrecht University, Den Burg, The Netherlands; 2 Department for Freshwater and Marine Ecology, University of Amsterdam, Amsterdam, The Netherlands; 3 Wageningen Marine Research, Den Helder, The Netherlands

**Keywords:** Sponge diet, Stable isotopes, Benthic–pelagic coupling, Saba Bank

## Abstract

Sponges are ubiquitous on coral reefs, mostly long lived and therefore adaptive to changing environmental conditions. They feed on organic matter withdrawn from the passing water and they may harbor microorganisms (endosymbionts), which contribute to their nutrition. Their diets and stable isotope (SI) fractionation determine the SI signature of the sponge holobiont. Little is known of spatio–temporal variations in SI signatures of δ^13^C and δ^15^N in tropical sponges and whether they reflect variations in the environment. We investigated the SI signatures of seven common sponge species with different functional traits and their potential food sources between 15 and 32 m depth along the S-SE and E-NE side of the Saba Bank, Eastern Caribbean, in October 2011 and October 2013. SI signatures differed significantly between most sponge species, both in mean values and in variation, indicating different food preferences and/or fractionation, inferring sponge species-specific isotopic niche spaces. In 2011, all sponge species at the S-SE side were enriched in d^13^C compared to the E-NE side. In 2013, SI signatures of sponges did not differ between the two sides and were overall lighter in δ^13^C and δ^15^N than in 2011. Observed spatio–temporal changes in SI in sponges could not be attributed to changes in the SI signatures of their potential food sources, which remained stable with different SI signatures of pelagic (particulate organic matter (POM): δ^13^C −24.9‰, δ^15^N +4.3‰) and benthic-derived food (macroalgae: δ^13^C −15.4‰, δ^15^N +0.8‰). Enriched δ^13^C signatures in sponges at the S-SE side in 2011 are proposed to be attributed to predominantly feeding on benthic-derived C. This interpretation was supported by significant differences in water mass constituents between sides in October 2011. Elevated NO_3_ and dissolved organic matter concentrations point toward a stronger reef signal in reef overlying water at the S-SE than N-NE side of the Bank in 2011. The depletions of δ^13^C and δ^15^N in sponges in October 2013 compared to October 2011 concurred with significantly elevated POM concentrations. The contemporaneous decrease in δ^15^N suggests that sponges obtain their N mostly from benthic-derived food with a lower δ^15^N than pelagic food. Average proportional feeding on available sources varied between sponge species and ranged from 20% to 50% for benthic and 50% to 80% for pelagic-derived food, assuming trophic enrichment factors of 0.5‰ ± sd 0.5 for δ^13^C and 3‰ ± sd 0.5 for δ^15^N for sponges. We suggest that observed variation of SI in sponges between sides and years were the result of shifts in the proportion of ingested benthic- and pelagic-derived organic matter driven by environmental changes. We show that sponge SI signatures reflect environmental variability in space and time on the Saba Bank and that SI of sponges irrespective of their species-specific traits move in a similar direction in response to these environmental changes.

## Introduction

The stable isotope (SI) approach is a widely used method to study sources of food and trophic transfer in food web studies. Application to sponges revealed a wide range in bivariate δ^13^C and δ^15^N signatures, illustrating interspecific differences in diet and isotopic niche space of sponges (Van Duyl et al., 2011; Freeman, Easson & Baker, 2014). In shallow water habitats, such as coral reefs, diets of sponges mainly comprise of pico- and nanophytoplankton, bacterioplankton, small detrital particles, dissolved organic matter (DOM) and metabolites provided by endosymbionts, which are hosted by various sponges (Erwin & Thacker, 2007; Freeman & Thacker, 2011; Maldonado, Ribes & Van Duyl, 2012). Therefore, coral reef sponges are considered as primary consumers living mainly on products of primary production, besides bacteria. Like other consumers, sponges are considered to reflect the SI signature of the ingested food mixture within 0–1‰ enrichment for carbon (δ^13^C), and 1.5–3.5‰ for nitrogen (δ^15^N) (Fry et al., 1982; Vander Zanden & Rasmussen, 2001; Fry, 2006). Endosymbiont communities also influence the SI signatures of their sponge hosts. Both sponge host and associated bacteria jointly determine the typical δ^13^C and δ^15^N signature of the sponge holobiont (Thacker & Freeman, 2012; Freeman, Easson & Baker, 2014). Sponges hosting phototrophic endosymbionts, which fix CO_2_ tend to have lower δ^13^C values than sponges without phototrophic endosymbionts and sponges hosting N_2_ fixing endosymbionts tend to have lower δ^15^N values (Fiore et al., 2010). Many sponges feed on DOM apart from plankton (Yahel et al., 2003; De Goeij et al., 2008; Mueller et al., 2014a; McMurray, Pawlik & Finelli, 2017; Hoer et al., 2018). It is widely recognized that sponges draw food from the pelagos to the benthos (benthic–pelagic coupling), but that sponges also ingest food derived from the benthos is less well-known (Southwell, 2007; Van Duyl et al., 2011; Rix et al., 2016a, 2016b).

The current phase shift from scleractinian coral to macroalgal, turf algal and cyanobacterial mat dominated communities (McCook, Jompa & Diaz-Pulido, 2001; Jackson et al., 2014; De Bakker et al., 2017) is considered to lead to an increased supply of DOM and suspended detrital material from the benthos to the reef overlying water (Haas et al., 2016). These non-calcifying benthic primary producers produce more DOM than corals per unit surface (Naumann et al., 2010a; Haas et al., 2010a, 2010b, 2011; Mueller et al., 2014b, 2016; Mueller, Meesters & Van Duyl 2017; Brocke et al., 2015). Moreover, there is increasing evidence that besides pelagic-derived food (mainly consisting of microbial loop food, e.g., pico- and nano(phyto)plankton), benthic-derived DOM may be an important food source for sponges (Van Duyl et al., 2011; Rix et al., 2016a, 2016b; Pawlik, Burkepile & Thurber, 2016). Additionally, suspended detrital material either derived from benthic or pelagic producers has also been identified as sponge food (Maldonado, Ribes & Van Duyl, 2012; McMurray et al., 2018).

The stable isotopic ratios (δ^13^C and δ^15^N) of benthic- and pelagic-derived food differ substantially (Fry et al., 1982; Raven et al., 1995). Pelagic primary producers (i.e., phytoplankton) in oligotrophic reef waters have usually lower δ^13^C values than benthic primary producers (Faganeli et al., 1988; France, 1995; Duarte, 1992; Van Duyl et al., 2011). Contrary, δ^15^N tends to be lower in benthic than in pelagic primary producers (Naumann et al., 2010b; Van Duyl et al., 2011; Kolasinski et al., 2016), particularly when N_2_ fixing takes place by turf algae, cyanobacterial mats or nitrogen fixing bacteria associated with various reef organisms (e.g., corals and sponges) (Lesser et al., 2007; Cardini et al., 2014; Den Haan et al., 2014). DOM released by phytoplankton, macroalgae and corals is generally assumed to closely reflect the SI signature of its source with little isotopic fractionation during formation and degradation (Williams & Gordon, 1970; Fry et al., 1998; Benner et al., 1997). Depending on sponge species, food from these pelagic and/or benthic sources appears to be assimilated in different proportions leading to distinct SI signatures (Van Duyl et al., 2011). For sponge diet changes to be reflected in the SI signature a 1–2 months delay related to the integration time for new source information is required (Freeman & Thacker, 2011; Simister et al., 2013). Variations in hydrographic conditions may be drivers of such changes (current directions and velocities, upwelling) in the tropics. Besides variations in water mass constituents flowing toward coral reefs, water turbulence and vertical mixing affect the availability of benthic and pelagic food for sessile organisms (Lowe & Falter, 2015). Fine scale hydrodynamic conditions vary in geographic space and time around reefs, with reefs differently exposed to incoming waves and currents and different water mass properties. Whether such variations are reflected in sponge diets is still unknown.

The aim of this study was to explore and explain variations in δ^13^C and δ^15^N of sponges on fore reefs with different orientation toward incoming currents and waves (space) over time on the Saba Bank. It was hypothesized that sponges with different functional traits along these stretches (sides) of the Bank receive food from different sources over time. We sampled food reaching sponges from open water transported by currents, and local food derived from benthic sources on the Saba Bank to estimate the contribution of pelagic- and benthic-derived food to their diet under different hydrodynamic conditions. To identify different water masses and infer the source of its constituents, we measured concentrations of organic matter and inorganic nutrients in space and time.

## Materials and Methods

Fieldwork was performed under the research permits #WSH/2011/1400 and RWS-2013/42681 issued by Rijkswaterstaat, Dutch Ministry of Infrastructure and Environment and Ministry of Economic Affairs.

### Location and hydrography

The Saba bank (17°25′N; 63°18′W) is a 2,200 km^2^ submerged carbonate platform as measured to the 200 m isobath in the northeastern Caribbean Sea. It is raised about 700–1,000 m above the general depths of the surrounding sea floor and lies 10–100 m below sea level with shallowest depths (∼ 10 m) along the E-NE fringes. The nearest island is Saba (ca five km from the NE border of the Bank), which is separated from the Saba Bank by a 600 m deep channel. The Saba Bank is fringed by relatively undisturbed (negligible local human impact) coral reefs along its S-SE and E-NE side with an average stony coral and sponge cover of 7.1% and 7.8%, respectively, and a benthic algal cover of approximately 50% in the 15–32 m depth range (Van Beek & Meesters, 2014). The mainly wind-driven Caribbean Current flushes and envelopes the Saba Bank from the SSE to the WNW. The source water for the Caribbean Current is from the equatorial Atlantic Ocean (North Equatorial, North Brazil and Guiana Currents) and enters the Caribbean basin mainly through the southern part of the Antillean Arc. The Saba Bank also receives oligotrophic water from the Antillean Current flowing into the Caribbean Basin directly from the Atlantic (Van Duyl, 2016).

**Figure 1:**
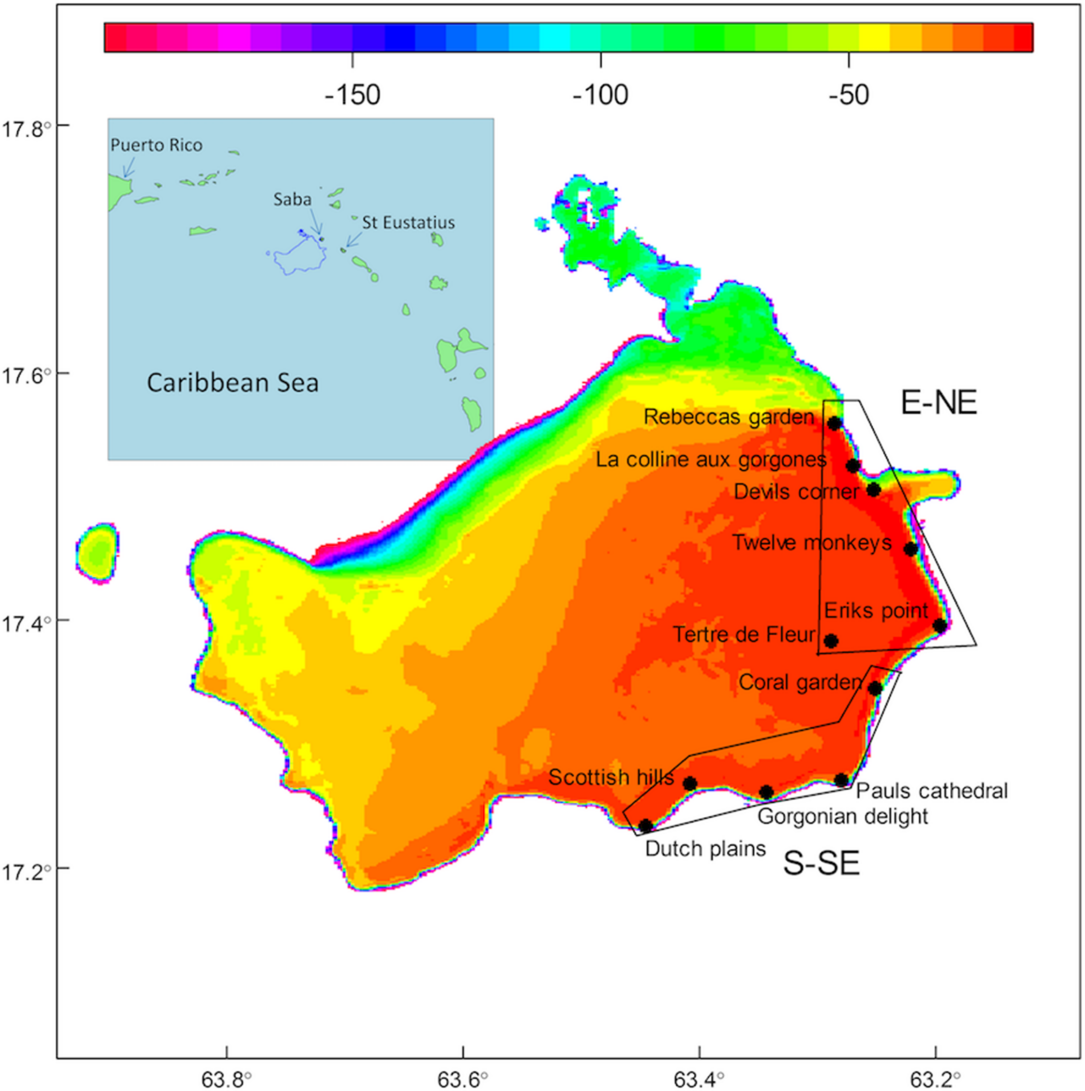
Map of the Saba Bank with inset. Map of the Saba Bank with depth contours (color scale in meter) and stations which were visited in October 2011 and October 2013. Fore-reef stations were in the 17–32 m depth range and the patch reef on top of the Saba Bank, Tertre de Fleur, was at 15 m depth. Stations Dutch plains until Coral garden were exposed to the S-SE and the rest of the stations had an E-NE exposition. Inset shows the position of the Saba Bank in the Caribbean Sea, with nearest islands Saba (∼5 km NE of the Saba Bank) and St. Eustatius (∼20 km East of the Saba Bank).

Fieldwork on the Saba Bank was conducted from the Caribbean Explorer II during two expeditions, the first from 22 to 28 October in 2011 and the second from 20 to 26 October in 2013. A total of 11 permanent coral reef stations, five stations along the S-SE side and six stations along the E-NE side of the bank were sampled, covering a distance of approximately 20 km along each side of the Bank (Fig. 1, see supplementary for coordinates and depth of stations). One site at the S-SE side (Dutch plains) was only sampled in 2013. Tidal amplitude is less than 10 cm per diurnal tidal cycle on the Bank, due to proximity of an amphidromic point (Kjerfve, 1981). Current speeds on the Bank in 2011 ranged from less than 0.25 up to 0.5 m/s (De Graaf, 2012). Water temperature varied between 29 and 30 °C in October 2011 and between 28 and 29 °C in October 2013.

### Collection of samples

Sponges and benthic macroalgae were collected by SCUBA diving at stations varying in depth between 15 and 32 m on gently sloping fore reefs. At each station, up to seven commonly occurring sponge species (one to three specimen per species) were collected belonging to three different functional groups varying in microbial abundance and chlorophyll-a content (Table 1). Chlorophyll-a content in sponges is indicative of the presence and abundance of phototrophic endosymbionts (Wilkinson, 1983; Erwin & Thacker, 2007; Freeman & Thacker, 2011). As representatives of benthic food sources two common brown macroalgal species *Dictyota* spp. and *Lobophora* spp. were collected at each station (one to two samples per species). On a few stations six or less sponge species were found. Particularly at the shallowest site (15–17 m, Tertre de Fleur) only four of the sponge species and one macroalgal species (*Dictyota* spp.) were found. At this station some other benthic algae, *Sargassum* and filamentous cyanobacteria were sampled for SI values. Particulate organic matter (POM) and dissolved (in)organic nutrients were sampled with a six L niskin bottle between zero and two m depth from the research vessel at each station. In 2011, two water samples were taken per station and in 2013 one water sample. The suspended particulate matter obtained from the surface water was considered to represent pelagic food for sponges. Vertical temperature and salinity profiles over reefs down to 35 m depth showed that the water column was well mixed (temperature nor salinity changed with depth).

**Table 1:** Sponge species. Collected sponge species with their functional traits, representing low microbial abundance sponges (LMA), high microbial abundance sponges with high chlorophyll-a (HMA-H) and low chlorophyll-a content (HMA-L). Selected sponge species were common on coral reefs of the Saba Bank (Thacker et al., 2010).

Sponge species	Functional trait	Chlorophyll-a (μg/g sponge tissue)	Reference
*Amphimedon compressa*	LMA	Negligible	Erwin & Thacker (2007) and Gloeckner et al. (2014)
*Callyspongia plicifera*	LMA	Negligible	Erwin & Thacker (2007) and Gloeckner et al. (2014)
*Aplysina cauliformis*	HMA-H	>125	Gloeckner et al. (2014)
*Xestospongia muta*	HMA-H	50–125	Erwin & Thacker (2007) and Gloeckner et al. (2014)
*Plakortis* spp.	HMA-H	50–125?	Gloeckner et al. (2014)
*Agelas conifera*	HMA-L	<50	Freeman, Easson & Baker (2014)
*Aiolochroia crassa*	HMA-L	<50	Erwin & Thacker (2007) and Gloeckner et al. (2014)

### Sample processing

Water samples for inorganic nutrients were filtered over 0.2 μm acrodisc syringe filters (25 mm diameter). Filtrate was collected in pony vials, which were stored in a deep freezer (−20 °C) until analysis. Samples were processed on an autoanalyzer (TRAACS) for PO_4_^3−^, NH_4_^+^, NO_x_, NO_2_^−^, (NO_x_ − NO_2_^−^ = NO_3_^−^). Water samples for DOM were also filtered over 0.2 μm acrodisc filters (25 mm diameter) and fixed with concentrated (38%) HCl (six drops for fixing 20 mL seawater) in combusted EPA vials (40 mL). Analysis of samples was done on a TOC-VCPN, Shimadzu analyzer yielding total and dissolved organic carbon (TOC, DOC) and total and dissolved nitrogen (TN, TDN). TDN minus dissolved inorganic nitrogen (DIN) (DIN = NH_4_ + NO_2_^−^ + NO_3_^−^) renders dissolved organic nitrogen (DON).

Suspended matter was collected on combusted glass fiber filters (Whatman GF/F, 47 mm diameter and 10 mm diameter). The volume of water filtered was noted and filters were folded with tweezers, wrapped in aluminum foil, labelled and stored in a freezer at (−20 °C). Subsequently, filters were fumed with acid vapor (HCl) in a desiccator to remove possible carbonates, and lyophilized.

Sponges and macroalgae were individually labelled and packed in aluminum foil immediately after the dive and stored in a freezer (−20 °C) until processing. Sponge and algal samples were treated with 2M HCl overnight on a shaking table to remove possible carbonates. This procedure was repeated if necessary. Afterward samples were washed with UltraPure water until a pH between 5 and 6 was reached. Subsequently, samples were lyophilized.

### Stable isotope analyses

For the SI analyses (δ^13^C and δ^15^N) homogenized organic matter portions of sponges and macroalgae (0.8–1 mg) and pieces of GF/F filters with suspended matter were transferred to tin cups, which were closed with tweezers. The C and N content of the dry weight and the isotope composition of the samples was determined on a Flash 2000 elemental analyzer coupled online with a Delta V Advantage-isotope monitoring mass spectrometer (Thermo Scientific, Waltham, Bremen, Germany) relative to standards (acetanilide, urea and casein). Values were normalized to acetanilide. C and N isotope ratios were expressed as δ^13^C to Vienna Pee Dee Belemnite standard and as δ^15^N to air. Standard error of measurements of standards was ∼0.15‰.

### Statistical analyses

Bayesian techniques were adopted to analyze SI data (Zuur, Hilbe & Ieno, 2013). Bayesian approaches use statistical distributions to characterize the uncertainties in the data. Bayesian methods were solely applied for analysis of SI data of sponges and potential organic food sources for sponges. Means and credible intervals of the separate isotopes have been calculated using R (R Core Team, 2017) and R-INLA (Rue, Martino & Chopin, 2009). All models included a Gaussian error distribution that was allowed to differ per species because initial data explorations indicated heterogeneity between sponge species. R-INLA is used frequently for analyses that include spatial and temporal correlations (Zuur, Ieno & Saveliev, 2017), but can be used for any type of (Bayesian) analysis (see extensive model specification on www.r-inla.org). For model selection Watanabe–Akaike information criteria was used.

For bivariate isotopic niche space identification all sponge data collected on the Bank in 2011 and 2013 were pooled per sponge species. The isotope niche area of each sponge species was determined with Stable Isotope Bayesian Ellipses in R (Jackson et al., 2011). The method estimated the standard ellipse area (SEA_c_) of the bivariate niche space of different sponges, after a correction for small sample size. Standard ellipses enveloped approximately 40% of the bivariate δ^13^C and δ^15^N data of the different sponge species. Means of isotopes with 95% credible intervals of different sponge species were also determined by Bayesian statistics using R-INLA.

Stable isotope samples of sponges and potential food sources collected at the S-SE were compared with samples at the E-NE of the Bank for 2011 and for 2013. Data analyses of δ^13^C and δ^15^N in sponges and potential food sources were conducted for each isotope separately because C and N metabolism and fractionation are not necessarily coupled (Maldonado, Ribes & Van Duyl, 2012).

An *isotope mixing model* was applied using R package SIAR, SI analysis in R, R package version 4.2 (Parnell & Jackson, 2013) to calculate the proportional contribution of benthic- and pelagic-derived food to the sponge diet. The model used the bivariate isotope data of sponges and potential food sources and fitted a Bayesian model to determine the dietary proportions of the different sources (Parnell et al., 2013; Phillips et al., 2014). Suspended POM was used as pelagic-derived food and fleshy benthic macroalgae (SI data of *Dictyota* spp. and *Lobophora* spp. were combined) as representative for benthic-derived food. An average trophic enrichment factors (TEFs) of primary consumers (sponges) of 0.5 ± sd 0.5% for δ^13^C and 3 ± sd 0.5 for δ^15^N (Peterson & Fry, 1987) was assumed. The model was run for sponges at S-SE and the E-NE sides of the Bank and for the different years with vague priors using default settings.

A *non-metric multidimensional scaling approach* (nMDS) was applied to characterize water samples on the bank and to investigate whether there were differences between sides of the bank or between years. Concentrations of PO_4_^3−^, NH_4_^+^, NO_2_^−^, NO_3_^−^, DIN, DIN/PO_4_^3−^ ratio, TOC, TON, DOC and DON and particulate organic carbon and nitrogen (POC, PON) and the ratio between POC and PON were used. The nMDS plot was generated after normalizing (scaling) values of environmental variables. Differences between samples were tested by Permanova (Anderson, 2001).

## Results

### Isotopic bivariate niche space of sponge species

**Figure 2:**
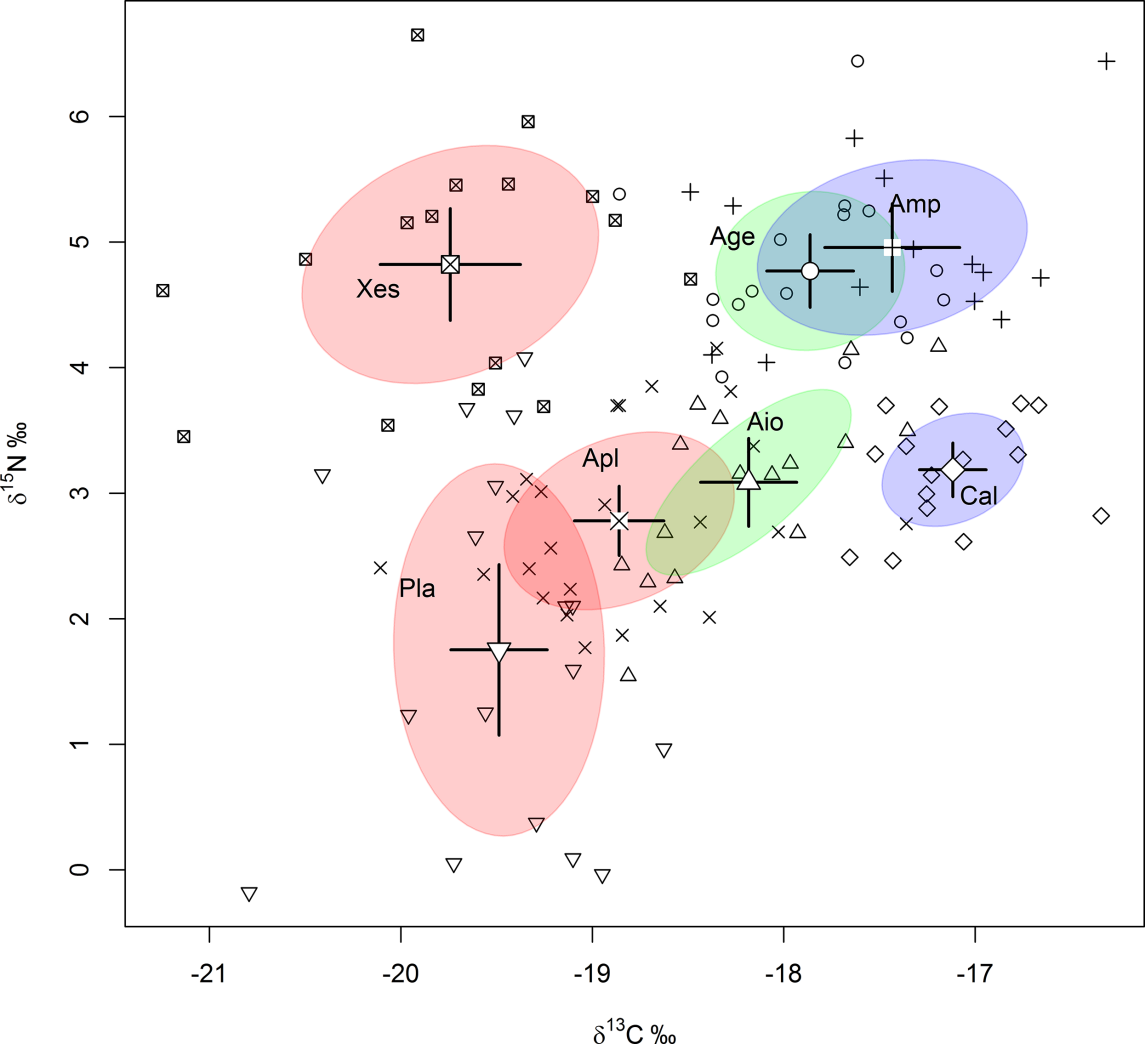
Sponge niche spaces. Isotopic bivariate niche space of seven sponge species on fore reefs of the Saba Bank, based on data collected in space and time. Standard ellipse areas of different sponge species were corrected for small sample size (SEAc). Bayesian means of different sponge species are shown within ellipses with the 95% credible intervals of the mean. HMA high chlorophyll-a sponges (HMA-H) are pink, HMA low chlorophyll-a sponges are green (HMA-L) and LMA sponges are purple. (+)Amp, *A. compressa*; (○)Age, *A. conifera*; (□)Xes, *X. muta*; (◊)Cal, *C. plicifera*; (▵)Aio, *A. crassa*; (x)Apl, *A. cauliformis*; (▽)Pla, *Plakortis* spp.

Combining data collected in this study over space and time, yielded ranges in δ^13^C and δ^15^N of sponges which showed considerable overlap between sponge species (Table 2). C/N ratios of sponges resembled those of bacteria with exception of the C/N ratio of *Plakortis* spp., which was surprisingly high for a High Microbial Abundance sponge (Table 2). Evident differences in the diet of sponges was best reflected in the isotopic bivariate niche spaces (Fig. 2). The placement, area and tilt of the SEAc around the different sponge species clearly varied (Fig. 2). HMA species were separated from Low Microbial Abundance species by differences in δ^13^C. Differences within functional groups (HMA-H, HMA-L and LMA) were mainly separated by differences in δ^15^N. The slight tilt of the ellipses of most sponge species indicated that SI ratios of C and N were mostly positively related.

**Table 2:** Sponge SI’s. Ranges and (Bayesian) means of δ^13^C and δ^15^N of stable isotope signatures and averages of C:N ratio’s (mol weight, raw data) of sponges with standard deviations. Sponges were collected between 15 and 32 m depth.

Sponges	*n*	Range ‰ δ^13^C	δ^13^C mean (sd)	Range ‰ δ^15^N	δ^15^N mean (sd)	Average C/N ratio (sd)
*Aplysina cauliformis*	24	−20.11 to −17.36	−18.86 (0.58)	1.77–4.16	2.78 (0.69)	4.7 (0.39)
*Plakortis* sp.	17	−20.79 to −18.63	−19.49 (0.52)	−0.18–4.08	1.75 (1.41)	8.2 (1.80)
*Xestospongia muta*	16	−21.24 to −18.49	−19.74 (0.72)	3.45–6.65	4.82 (0.89)	4.9 (0.49)
*Agelas conifera*	17	−18.86 to −17.16	−17.86 (0.46)	3.92–6.44	4.77 (0.60)	4.3 (0.27)
*Aiolochroia crassa*	16	−18.85 to −17.19	−18.18 (0.51)	1.54–4.17	3.09 (0.70)	4.9 (0.32)
*Amphimedon compressa*	14	−18.49 to −16.31	−17.43 (0.67)	4.04–6.44	4.96 (0.66)	4.1 (0.52)
*Callyspongia plicifera*	16	−17.66 to −16.34	−17.12 (0.34)	2.46–3.71	3.19 (0.42)	3.6 (0.05)

### Spatial and temporal variation in SI signatures of sponges

**Figure 3:**
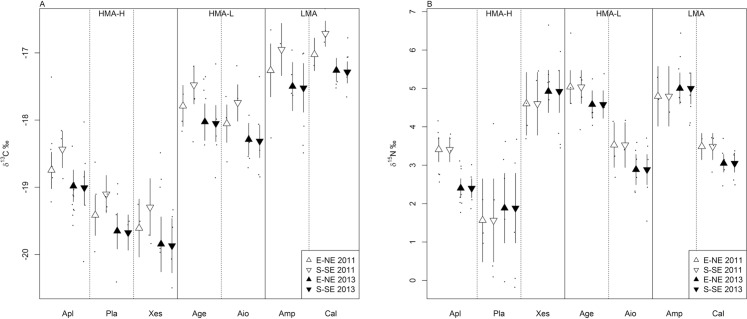
Variation in δ^13^C (A) and δ^15^N (B) of different sponge species along the S-SE and E-NE side of the Saba Bank in 2011 and 2013. Means and 95% credible intervals are based on Bayesian statistics. Black dots represent the raw data of δ^13^C (A) and of δ^15^N (B), respectively, of the different sponge species. Sponges are grouped according to their functional traits (HMA-H, HMA-L and LMA). For sponge species abbreviations see Fig. 2.

We found that δ^13^C of the different sponge species varied in space and time, and that δ^15^N only varied in time on the Saba Bank (Figs. 3A and 3B). The δ^13^C values of the sponges were best described by a model that included species, sampling year, side of the bank, as well as an interaction between these latter two variables:

$${{\rm{\delta }}^{13}}{{\rm{C}}_{{\rm{sponge}}}}\sim{\rm{Species}} + {\rm{Side}} + {\rm{Year}} + {\rm{Side}}:{\rm{Year}}$$

The best model for values of δ^15^N included only the main effects, “species” and “year of sampling”:

$${{\rm{\delta }}^{15}}{{\rm{N}}_{{\rm{sponge}}}}\sim{\rm{ Species}} + {\rm{Year}}$$

“Species” refers to sponge species, “Side” to the E-NE facing or S-SE facing side of the Saba Bank and “Year” to October 2011 or October 2013. Raw data for Figs. 3A and 3B are presented in Supplementary Information.

Sponge species was the main driver of variations in δ^13^C and δ^15^N. In space and time, however, the δ^13^C of sponge species varied in concert irrespective of their different functional traits (Fig. 3A). Mean δ^13^C was higher at the S-SE than the E-NE side in 2011 and decreased from 2011 to 2013 in all sponge species. An evident interaction effect between Side and Year for variations in δ^13^C was observed, implying that spatial differences in δ^13^C in sponges between sides with different orientation toward incoming currents and waves occurred, but varied in time. In 2011, the δ^13^C signatures in sponges was on average 0.31‰ lower at the E-NE side than at the S-SE side (mean 0.31, 95% credible interval 0.04–0.59). From 2011 to 2013 δ^13^C signatures decreased in all sponges species on average with 0.23‰, but sponges at the S-SE side decreased even further than at the E-NE side with 0.34‰ (median 0.34, 95% credible interval −0.67 to −0.01). This decrease of δ^13^C in sponges at the S-SE side compensated the difference that existed in 2011 between sides, making sides again comparable in 2013.

Overall the δ^15^N in sponges (Fig. 3B) showed an evident temporal effect with a mean decrease of 0.51‰ (95% credible interval 0.27–0.75) from 2011 to 2013. Spatial variations were not found and variations in δ^15^N of sponges in time were not consistent for species within HMA-H and LMA groups. Only *A. cauliformis*, *A. conifera* (Age), *A. crassa* and *C. plicifera* showed a clear decrease in δ^15^N from 2011 to 2013 with little or no overlap of 95% credible intervals. The mean δ^15^N of other sponge species (*Plakortis* spp., *X. muta* and *A. compressa*) did not clearly change in time. No relations were found between sponge SI signatures and depth.

### Stable isotope signatures of potential food sources for sponges

Stable isotope signatures of benthic genera of fleshy brown algae and cyanobacterial mats largely overlapped and were evidently higher in δ^13^C and lower in δ^15^N than the pelagic-derived food, POM (Table 3). *Sargassum* spp. and cyanobacteria were only collected at up to three stations. Therefore, only SI data of *Dictyota* spp. and *Lobophora* spp. were used and pooled as benthic-derived food representative, and compared with the pelagic food source (POM). SI signatures of suspended POM (δ^13^C: −24.91‰ [95% credible interval −24.20‰ to −25.63‰]; δ^15^N: 4.33‰ [95% credible interval 3.66–4.99‰]) clearly differed from the benthic source (δ^13^C: 15.38‰ [95% credible interval −14.69‰ to−16.07‰] and δ^15^N: 0.80‰ [95% credible interval 0.47–1.14‰]), with lower mean δ^13^C (9.5‰ lower) and higher δ^15^N values (3.5‰ higher). The C/N ratio of pelagic POM was lower compared to that of benthic macroalgae, but overlapped with benthic cyanobacteria (Table 3). No differences in geographic space or time were observed for δ^13^C and δ^15^N SI signatures of benthic food sources. The same was found for the δ^13^C in pelagic food (POM). For δ^15^N in POM insufficient data were available to test its variation in space and time (only one observation in 2011 and seven in 2013). Values obtained in 2013, however, did not show spatial variation and covered the single value available for 2011 assuming that also temporal variation was negligible.

**Table 3:** Benthic algae and POM. Ranges and (Bayesian) means of δ^13^C and δ^15^N of stable isotope signatures of benthic algae, benthic cyanobacterial mats and particulate organic matter (POM) and averages of C:N ratio’s (mol weight, raw data) with standard deviations. Benthic algae were collected between 15 and 32 m depth. POM was obtained from surface water (0 to 2 m).

Benthic algae and POM	*n*	Range in ‰ δ^13^C	δ^13^C mean (sd)	Range in ‰ δ^15^N	δ^15^N mean (sd)	Average C/N ratio (sd)
*Dictyota* spp.	15	−18.44 to −15.04	−16.60 (1.17)	−0.26–1.19	0.47 (0.60)	18.7 (4.08)
*Lobophora* spp.	16	−18.97 to −11.21	−14.23 (2.10)	−0.19–3.38	1.12 (0.93)	33.6 (6.00)
*Sargassum* spp.	3	−19.50 to −14.62	−16.31 (2.36)	−0.41–2.52	1.31 (1.32)	35.4 (3.80)
Filamentous benthic cyanobacteria	1		−15.07		0.00	8.1
POM in surface water	19.8	−27.67 to −17.78	−24.91 (1.77)	2.92–6.25	4.33 (1.24)	12.9 (5.52)

### Source partitioning

The proportional contribution of pelagic- and benthic-derived food to the diet of the seven different sponge species revealed that based on the assumed TEFs of C (0.5‰ ± sd 0.5) and N (3.0‰ ± sd 0.5), diets varied depending on sponge species from 51%/49% (LMA sponge *C. plicifera*) to 21%/79% (HMA-H sponge *X. muta*) with regard to benthic/pelagic feeding. LMA sponges appeared to rely more on benthic-derived food than HMA sponges. Five (*X. muta*, *A. conifera*, *A. crassa*, *A. compressa* and *C. plicifera*) of the seven sponge species were estimated to consume relatively more benthic food at the S-SE side than at the E-NE side in 2011 based on the means of the 95% credibility internals (Table 4). In 2013, *X. muta* and *Plakortis* spp. were estimated to consume on average relatively less benthic food and thereby more pelagic-derived food than their conspecifics in 2011. Results showed that average shifts in benthic–pelagic feeding of ∼1 (*A. crassa*) to ∼13% (*A. compressa*) would be sufficient to cover the recorded differences in bivariate isotope signatures of sponges in space in 2011. To cover changes in time in sponge SI’s along the S-SE and E-NE side, shifts of up to 19% (*Plakortis* spp.) in benthic–pelagic feeding were estimated. It should be noted here that the 95% credibility internals were large (Table 4), which made it impossible to assess evident differences in proportional feeding of sponges between different sides of the Bank in 2011 and between years.

**Table 4:** Contribution of benthic food to sponge diet. Bayesian means with 95% credibility internals (between brackets) of benthic food contribution (%) to the diet of different sponge species in space and time according to the Stable Isotope mixing model (Parnell et al., 2013). Complementary % to reach 100% is ascribed to pelagic food (not shown).

Sponges	S-SE 2011 (%)	E-NE 2011 (%)	S-SE 2013 (%)	E-NE 2013 (%)
*Aplysina cauliformis*	38.7 (20–57)	40.7 (29–53)	45.2 (26–64)	40.5 (28–53)
*Plakortis* spp.	36.4 (14–58)	53.0 (15–91)	30.3 (11–50)	33.9 (15–53)
*Xestospongia muta*	29.1 (0–59)	25.7 (4–47)	20.7 (7–34)	22.9 (5–41)
*Agelas conifera*	37.6 (17–58)	35.5 (10–61)	34.2 (18–51)	39.0 (24–54)
*Aiolochroia crassa*	43.3 (15–71)	42.7 (24–61)	44.3 (30–58)	44.2 (32–56)
*Amphimedon compressa*	48.8 (5–93)	36.5 (2–71)	37.4 (21–45)	45.2 (30–61)
*Callyspongia plicifera*	50.9 (37–65)	49.1 (4–94)	51.2 (40–62)	49.1 (37–61)

### Water mass nutrient concentrations

**Figure 4:**
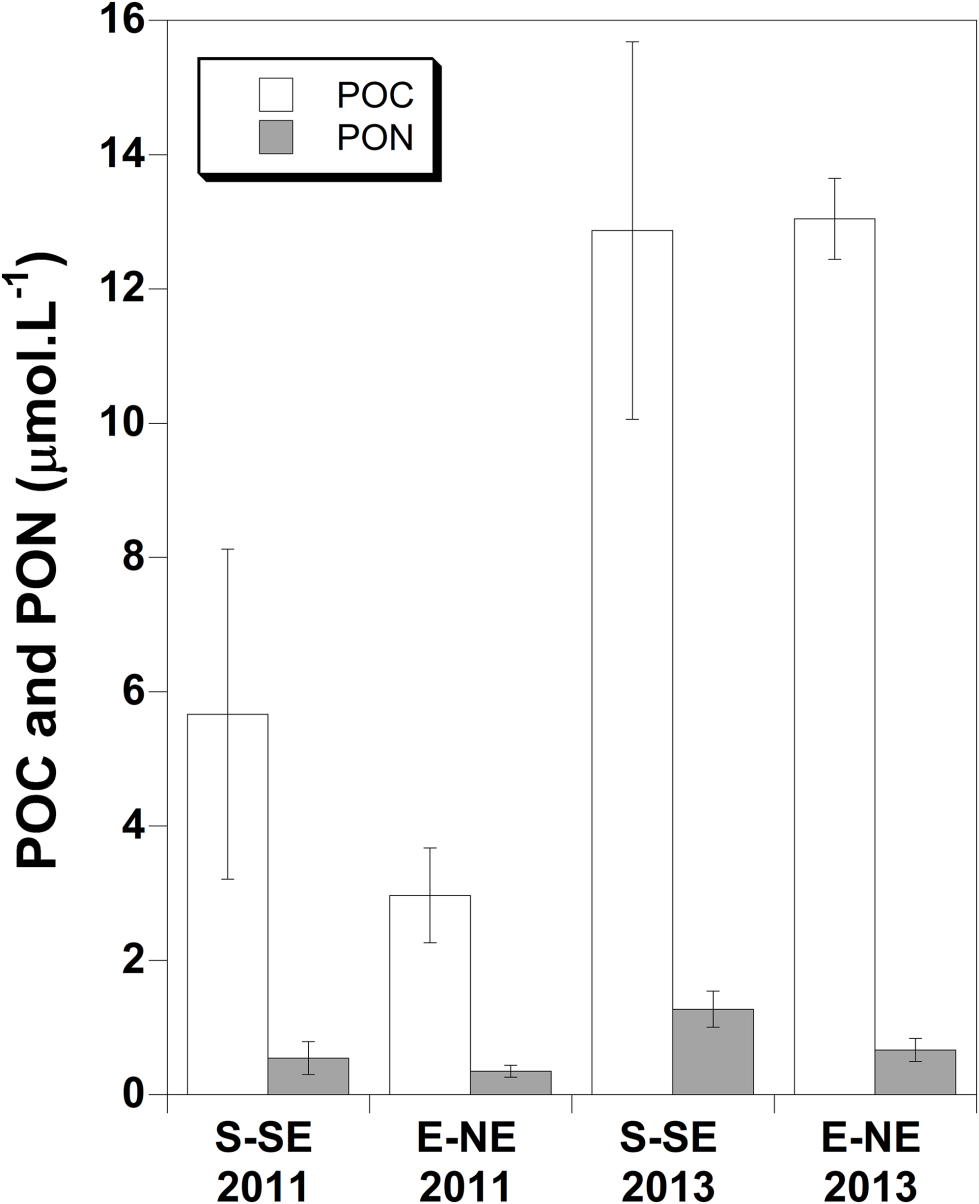
POC and PON concentrations on the Saba Bank. Variations in particulate organic carbon (POC) and nitrogen (PON) concentrations in surface water along the S-SE and E-NE side of the Saba Bank in 2011 and 2013 with standard deviations.

**Figure 5:**
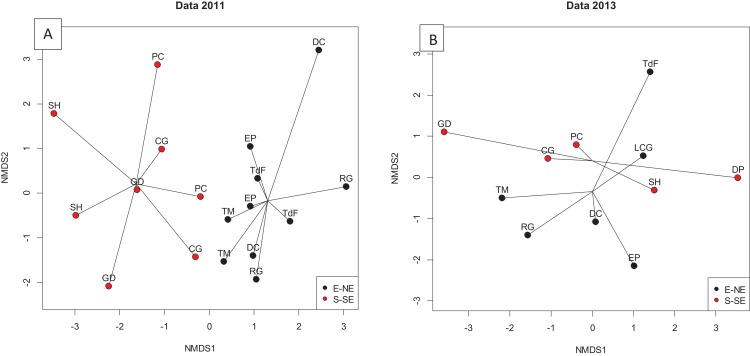
Water mass characterization. Numerical dimensional scaling plots with water mass differences on basis of the following variables: average PO_4_^3−^, NH_4_^+^, NO_2_^−^, DIN, DIN/PO_4_^3−^, ratio, DOC, DON, TOC, TON, POC and POC/PON ratio at different sides of the Saba Bank (S-SE and E-NE) in (A) October 2011 and (B) October 2013. Samples along the S-SE side were collected at stations DP, SH, GD, PC, CG and along the E-NE side at EP, TdF, DC, TM, LCG and RG (i.e., abbreviations of station names, see Fig. 1 or Supplemental Data for full names of locations).

In October 2013, POM concentrations in surface waters were significantly higher along both sides of the Bank than in 2011 (Fig. 4). POC/PON ratios differed between sides in 2013 with a ratio of 20.5 ± sd 4.8 at the E-NE and 10.2 ± sd 2.0 at the S-SE side. The latter ratio was comparable to the ratios found in 2011 along both sides (10.1 ± sd 0.8 and 9.2 ± sd 0.7 at the S-SE and E-NE side, respectively). Concentrations of all other organic and inorganic nutrient variables were not significantly different between sides in 2011 or in 2013. However, taking all nutrient variables in surface water into account (except the ones showing high autocorrelation, e.g., NO_3_^−^, TN, TOC), a clear difference in water mass constituent proportions (NH_4_^+^, NO_2_^−^, PO_4_^3−^, DOC, DON, POC concentrations and DIN/PO_4_^3−^ and POC/PON ratio’s) between the S-SE and E-NE side was found in 2011 (Fig. 5A). Testing the distance matrix ∼ side of the nMDS results, yielded a *p*-value of <0.001 (Permanova test). The difference in water masses between sides in 2011 was characterized by higher average DIN (notably NO_3_^−^), DOC, POC and PON concentrations and DIN/PO_4_^3−^ ratio along the S-SE than E-NE side of the Bank, and lowest average PO_4_^3−^ values at the S-SE side in 2011. Particularly the higher concentrations of NO_3_^−^ of 0.197 ± sd 0.15 at the S-SE vs 0.111 ± sd 0.04 μmol/L at the E-NE (Fig. S1) and lower concentrations of PO_4_^3−^ (∼0.013 μmol/L, Fig. S2) at S-SE side in combination with higher DOC concentrations (Fig. S3) and DIN/PO_4_^3−^ ratios (Fig. S4) than the E-NE side pointed to a reef signal in the water mass in 2011. In 2013, the water mass at the S-SE was not significantly different from the water mass at the E-NE side (Fig. 5B, Permanova test, *p* = 0.596). See Supplemental Information for concentrations of all nutrients.

## Discussion

We show that carbon and nitrogen SI compositions of sponges on the Saba Bank vary in geographic space and through time. Trajectories of change in SI signatures were comparable between most sponge species irrespective of different functional traits. Variations in sponge δ^13^C signature in geographic space concurred with differences in water mass constituents. Differences in the composition of these constituents suggest different exposure times to reef waters due to differences in hydrodynamic conditions between the E-NE and S-SE side of the Bank in 2011. Observed lower δ^13^C and δ^15^N values of sponges in 2013 compared to 2011 was suggested to be induced by an increase in pelagic food, which might have influenced the proportion of C and N absorbed from pelagic- and benthic-derived food by sponges differently.

### Variation in bivariate isotopic niche space in sponges

Sponge species in this study showed ranges in SI signatures, which agree well with ranges for similar sponge species in the Caribbean (Southwell, 2007; Weisz et al., 2007; Fiore, Baker & Lesser, 2013; Freeman, Easson & Baker, 2014). This might imply that bivariate isotope niche space of these sponge species persisted in space and time and that sponges maintained their trophic position relative to other sponge species. The placement of the niche spaces (SEAs_c_) in bivariate plots showed clear differences between sponge species indicating distinct diets. Larger niche spaces suggest that species have a broader access to food sources and might be more apt to maintain themselves under varying trophic conditions than species with relatively small niche spaces (Freeman, Easson & Baker, 2014, 2016). The observation that the ellipses of most sponges slightly tilted to the right indicate that the δ^13^C and δ^15^N tended to follow comparable trajectories of change irrespective functional traits (HMA vs LMA sponges). This suggested that the basic sponge metabolism overruled the contribution of its endosymbionts.

Interestingly, the dichotomy of HMA and LMA sponges (Gloeckner et al., 2014) was mainly covered by differences in δ^13^C. HMA-H sponges had lower δ^13^C values than LMA sponges. This difference might be attributed to the presence of phototrophic endosymbionts, which preferably fix inorganic ^12^C over ^13^C (Freeman, Easson & Baker, 2014) and supply HMA-H sponges with photosynthates. Niche spaces of HMA-L sponges were found to be in between HMA-H and LMA sponges, which suggest that they rely more on external food sources and to a lesser part on food supplied by their endosymbionts Patterns for δ^15^N were unresolved with respect to HMA–LMA dichotomy, suggesting that N might be obtained from a different source and/or that fractionation varies between sponge species. The division we found between the low <3.5 and high >4‰ δ^15^N values in HMA sponges has also been reported by others, although at a slightly different separation of the low δ^15^N group (Weisz et al., 2007; Mohamed et al., 2010). The LMA group mainly resides in the high δ^15^N group (Freeman, Easson & Baker, 2014), although in our study the LMA sponge *C. plicifera* was part of the low group. Complex biochemical N cycling pathways involving different N fractionation have been reported for both HMA (Weisz et al., 2007; Southwell et al., 2008; Fiore et al., 2010; Fiore, Baker & Lesser, 2013) and LMA sponges (Schläppy et al., 2010; Cardoso et al., 2013; Diaz & Ward, 1997; Jiménez & Ribes, 2007; Southwell, Popp & Martens, 2008; Southwell et al., 2008; Van Duyl et al., 2008). Nitrification and other microbial metabolic pathways (e.g., denitrification, anammox, N_2_ fixation) probably influenced the δ^15^N signature of the sponge holobiont (Weisz et al., 2007; Southwell et al., 2008; Mohamed et al., 2010). Nitrification and denitrification increased the δ^15^N values in sponges (Southwell et al., 2008), while sponges harboring N_2_ fixing microbes tended to decrease in δ^15^N (Wilkinson & Fay, 1979; Southwell, Popp & Martens, 2008; Southwell et al., 2008; Taylor et al., 2007; Weisz et al., 2007; Mohamed et al., 2008; Ribes et al., 2015). To what extent N_2_ fixation contributed to the differences in δ^15^N of the sponge holobiont is still questionable (Southwell et al., 2008) and might depend on ambient nutrient conditions. Overall these and our results suggest that the carbon and nitrogen incorporated in the sponge holobiont were not necessarily retrieved from the same source.

### Variations in sponge SI signature in space and time

To date it is still poorly understood to what extent hydrodynamic conditions in geographic space and/or time affect variations in SI signature of different sponge species. Variations in space of δ^13^C and δ^15^N of corals, macroalgae, suspended matter, (in)organic nutrients and sponges on coral reefs have mostly been described in relation to onshore–offshore distances and depth (Risk, Sammarco & Schwarcz, 1994; Risk et al., 2009; Southwell, 2007; Lapointe et al., 2010; Wyatt et al., 2013). On the Saba Bank the distance from shore did not play a role and the depth gradient from 15 to 32 m, in which sponges were collected, did not influence the spatial variation in SI signatures of sponges on the Saba Bank. Nevertheless, in 2011 clear differences in SI signatures were found between the E-NE and the S-SE sides of the Bank. All sponges irrespective of species or functional traits had higher δ^13^C values at the S-SE side than at the E-NE side in 2011. Each species was on average (raw data) 0.01–0.64‰ higher in δ^13^C than its conspecifics at the other side. With Bayesian statistics the best estimate for all sponges yielded a difference of 0.31 (95% credible interval of 0.04–0.59) in δ^13^C between sides. This was comparable to ranges found for δ^13^C signatures of *X. muta* between different sites in the NE and W Caribbean (Fiore, Baker & Lesser, 2013; Freeman, Easson & Baker, 2014), but smaller than differences found for onshore–offshore gradients of up to 5‰ (Southwell, 2007). The fact that all seven sponge species had higher δ^13^C values at the S-SE-side than the E-NE side implied that this phenomenon was likely to be caused by general differences in organic matter supply of pelagic and/or benthic sources, rather than by species-specific functional traits, such as the presence and abundance of (phototrophic) endosymbionts. As sponges only differed in δ^13^C, but not in δ^15^N signatures, it further appeared that N-metabolism of sponges, irrespective of sponge species, was not affected by space on the Saba Bank. The effect of spatial differences in hydrodynamic conditions concerning pelagic food supply were apparently more important for the δ^13^C than for the δ^15^N in sponges, which is in accordance with other studies (Southwell, 2007; Freeman, Easson & Baker, 2014).

Spatial differences in δ^13^C of sponge species were only found in 2011, but not in 2013. This indicated that the difference was not determined by the fixed position of the Saba Bank toward incoming currents and waves. The environmental conditions were apparently different between sides (S-SE and E-NE side) in 2011 (see below), which lasted over a long enough period for sponges to obtain a distinct δ^13^C signal. Integration time for a distinct signal has been estimated on 1–2 months (Freeman & Thacker, 2011; Simister et al., 2013). Thus, it is likely that divergent environmental conditions led to the increased δ^13^C signature in sponges at the S-SE side and not at the E-NE side, where variations in δ^13^C in time were smaller than at the S-SE side.

Time affected both the δ^13^C and the δ^15^N values of sponges on the Saba Bank. The sponge SI signatures decreased between 2011 and 2013 with 0.23‰ for δ^13^C and with 0.51‰ for δ^15^N. That decreases in δ^13^C in time at the S-SE side (0.34‰) were larger than at the E-NE side suggested that the uptake of pelagic-derived food was higher, purportedly due to an increased supply rate at the S-SE than E-NE side preceding our sampling in 2013. The variability in hydrodynamics in time might have been larger at the S-SE than E-NE side of the Bank supporting the higher fluctuation in δ^13^C at the S-SE side. Comparable trajectories of excursions of bulk δ^13^C and δ^15^N in sponges in time were also observed by Simister et al. (2013). They (ibid) reported several significant excursions from the mean δ^13^C and δ^15^N values in the LMA sponges *Ancorina alata* and *Tethya stolonifera* without a significant change over a 2-year period. Interestingly the trajectories of the excursions were comparable between the sponge species irrespective species-specific differences in SI signatures, which is in accordance with our SI changes for most sponge species in space and time. Comparable patterns of change in the δ^13^C and δ^15^N of most sponges appeared particularly driven by temporal variation in dietary food sources.

### Food sources contributing to sponge SI signatures

Contrasting to the spatio–temporal variations in sponge SI signatures, SI signatures of pelagic POM and the benthic macroalgae, as well as their difference remained stable in both space and time. Pelagic-derived food was ∼9.5‰ lower in δ^13^C and ∼3.5‰ higher in δ^15^N compared to these values in benthic-derived food. Comparable differences in SI signatures between pelagic and benthic primary producers in coral reef ecosystems have been reported by others (Granek, Compton & Phillips, 2009; Van Duyl et al., 2011; Wyatt et al., 2013; Kürten et al., 2014; Kolasinski et al., 2016). We therefore, propose that shifts in benthic vs pelagic feeding occurred to explain variations in SI signatures in space and time. On average 25–50% of the sponge diets on the Saba Bank consisted of benthic-derived food. The HMA-H sponges *X. muta* relied the least on benthic food (25% ± sd 4%) and the LMA sponge *C. plicifera* the most (50% ± sd 1%). Shifts of less than 10% in the diet of sponges (Table 4) would be sufficient to explain the recorded spatio–temporal patterns in most sponge bivariate SI signatures. Such shifts were likely triggered by local environmental (hydrodynamic) conditions influencing the availability of benthic- and pelagic-derived food.

### Effect of environmental conditions on stable isotopic signatures in sponges

The spatial enrichment of δ^13^C in sponges in 2011 along the S-SE side of the Bank concurred with a small, but significant difference in water mass constituent proportion between sides (Fig. 5A). Although upwelling and different water mass intrusions occur along the Saba Bank and Saba (Van Duyl, 2016; De Nooijer & Van Heuven, 2016; Van Heuven et al., submitted), no clear indications were found to support that such events occurred during the sampling in October 2011 and October 2013. Therefore, water masses at the different sides of the bank might have come from different sources or the retention time of the water on the fore reefs along the S-SE and E-NE side of the Bank differed. The general trend is that the main surface flow approaching the Saba Bank comes from the SSE and slightly bends toward more westerly directions over and along the Bank to continue to the NW (Gordon, 1967). However, source water reaching the Bank and flow patterns along and over the Bank can be highly variable (Andrade & Barton, 2000). Model simulations of source, track and speed of water parcels reaching the Saba Bank in October 2011 and October 2013 were quite different (A. Candy, 2017, unpublished data). This might also have affected water mass constituents and SI signatures of sponges found between sides and years at the Saba Bank. Increased average NO_3_^−^ concentrations, decreased PO_4_^3−^ and slightly enhanced DOM concentrations pointed to an elevated reef benthic influence on water mass constituents at the S-SE compared to the E-NE side in 2011. This implied that the retention time of water might have been longer on the reefs along the S-SE than E-NE side of the Bank. Sponge and coral holobionts are known for their potential to nitrify leading to increased nitrate concentrations above reefs (Webb et al., 1975; Corredor et al., 1988; Wafar, Wafar & David, 1990; Diaz & Ward, 1997; Gast et al., 1999; Southwell et al., 2008). Also increased DOC concentrations have been reported for reef overlying waters (Van Duyl & Gast, 2001; Dinsdale et al., 2008; Mueller et al., 2014b; Mueller, Meesters & Van Duyl, 2017). Sponges obtaining food from this water mass might therefore, have been higher in δ^13^C than sponges at the E-NE side in 2011. The fact that the δ^15^N signatures did not differ spatially in 2011 nor in 2013 might indicate that sponges obtain N for a large part from benthic-derived organic matter. Organic N requirements of sponges are high considering the low C:N ratio of most sponges in this study and the generally high inorganic N exudation rates (Maldonado, Ribes & Van Duyl, 2012).

The decrease of δ^13^C in sponges from 2011 to 2013 is most likely driven by higher POC concentrations in October 2013 than in October 2011 which might have affected the proportional feeding of benthic- and pelagic-derived food in favor of pelagic-derived food. The δ^13^C in phyto-, bacterioplankton comprising most of the POM is much lower than in benthic-derived organic matter, which might have been reflected in the SI signature of sponges at the S-SE as well as the E-NE side in 2013. That several sponge species had lower δ^15^N values (*A. cauliformis*, *A. conifera* and *C. plicifera*) in 2013 than 2011 is more difficult to explain considering the fact that plankton derived PON was higher in δ^15^N (mean δ^15^N is 4.3‰) compared to benthic-derived food (0.8‰). It suggests that also feeding on benthic-derived nitrogen was intensified by increasing the proportion of benthic N in the diet of these sponges. Additionally, the lower C/N ratio of POM along the E-NE side in 2013 than in 2011 potentially contributed to this proportional shift. Furthermore, it is well-known that cyanobacterial mats and turf algae on coral reefs fix N_2_ and that δ^15^N signatures of cyanobacterial mats are lower than of other benthic primary producers (Table 3; Kayanne et al., 2005; Kolasinski et al., 2016). Released products from these mats contain N compounds lower in δ^15^N than N released by macroalgae or pelagic primary producers (Kayanne et al., 2005; Thibodeau et al., 2013; Brocke et al., 2015). In the Caribbean significant increases in cyanobacterial mat cover on coral reefs were reported from less than 10 to more than 20% since ∼1990 (De Bakker et al., 2017). Such shifts in cover might have occurred on the Saba Bank as well, considering the fact that in October 2013 ca 30% of the cover of benthic primary producers and 20% of total cover on reefs of the Saba Bank was occupied by cyanobacterial mats (Wiltink, 2016). In case sponges feed on this cyanobacterial mat derived nitrogen, it is likely that δ^15^N in their bulk tissue further decreased in time. It is evident that various nitrogen sources contributed to the observed patterns of δ^15^N in sponges in time.

## Conclusion

Bivariate SI signatures of sponges (δ^13^C, δ^15^N) on coral reefs of the Saba Bank differed between species with different functional traits. Orientation of reefs toward incoming currents and waves (space) influenced the variation of δ^13^C in sponges but not the δ^15^N. Bulk δ^13^C and δ^15^N in sponges moved in a comparable direction in time, while the δ^13^C appeared more responsive to changes in pelagic food concentrations than the δ^15^N. The variable supply of pelagic-derived organic carbon to sponges and the accumulation of benthic-derived food in reef overlying waters was ascribed to variations in hydrodynamic conditions in time and space (currents and retention time of water over reefs). Variations in SI signatures of sponges were most likely realized by mainly unselective shifts in proportional feeding on pelagic- and benthic-derived food sources, which differed significantly in SI composition. Shifts in diet were likely influenced by spatial differences in retention time of water over reefs and concentration of pelagic food relative to benthic-derived food.

## Supplemental Information

10.7717/peerj.5460/supp-1 Supplemental Information 1 Fig. S1. Nitrate concentrations on the Saba Bank. Variations in inorganic nitrate (NO_3_^−^) in surface water along the S-SE and E-NE side of the Saba Bank in 2011 and 2013 with standard deviations.

10.7717/peerj.5460/supp-2 Supplemental Information 2 Fig. S2. Phosphate concentrations on the Saba Bank. Variations in soluble reactive phosphate (PO_4_^3−^) in surface water along the S-SE and E-NE side of the Saba Bank in 2011 and 2013 with standard deviations.

10.7717/peerj.5460/supp-3 Supplemental Information 3 Fig. S3. Dissolved organic carbon concentrations on the Saba Bank. Variations in dissolved organic carbon (DOC) in surface water along the S-SE and E-NE side of the Saba Bank in 2011 and 2013 with standard deviations.

10.7717/peerj.5460/supp-4 Supplemental Information 4 Fig. S4. DIN/PO_4_^3−^ ratio on the Saba Bank.

10.7717/peerj.5460/supp-5 Supplemental Information 5 Saba Bank raw sponge stable isotope and nutriënt data. (Sheet Stations) Names and positions of stations sampled on the Saba Bank in October 2011 and October 2013.(Sheet Sponges SI) Stable isotope signatures of sponges collected on the Saba Bank in October 2011 and October 2013. Position of station numbers are presented on sheet Stations.(Sheet Macroalgae SI) Stable isotope signatures of benthic primary producers collected on the Saba Bank in October 2011 and October 2013. Position of station numbers are presented on sheet Stations. (Sheet POM SI) Stable isotope signatures of particulate organic matter (POM) collected in surface water on the Saba Bank in October 2011 andOctober 2013. Position of station numbers are presented on sheet Stations.(sheet Nutriënt concentrations) Organic and inorganic nutrient concentrations in surface water at stations sampled in October 2011 and 2013 on the Saba Bank. In 2011 replicate water samples were analysed. PO_4_^3−^ is soluble reactive phosphate; Sum of ammonium (NH_4_^+^) nitrite (NO_2_^−^) and nitrate (NO_3_^−^) represents dissolved inorganic nitrogen (DIN); DOC is dissolved organic carbon; DON is dissolved organic nitrogen obtained by distracting DIN from total dissolved nitrogen (TDN); TOC is total organic carbon; TON is total organic nitrogen obtained by distracting DIN from total nitrogen (TN); POC is particulate organic carbon; PON is particulate organic nitrogen. Position of station numbers are presented on sheet Stations.

## Additional Information and Declarations

### Field Study Permissions

The following information was supplied relating to field study approvals (i.e., approving body and any reference numbers): Research permits for conducting research on the Saba Bank in 2011 and 2013 were issued by Rijkswaterstaat, Dutch Ministry of Infrastructure and Environment and Ministry of Economic Affairs (No. WSH/2011/1400 and No. RWS-2013/42681).

### Data Availability

The following information was supplied regarding data availability: The raw data are provided in a Supplemental File.
