# Supplementary figures and images for: Spatio–temporal variation in stable isotope signatures (δ13C and δ15N) of sponges on the Saba Bank
Source: PeerJ. 2018 Aug 14;6:e5460. doi: 10.7717/peerj.5460 (PMC6097495; doi:10.7717/peerj.5460)

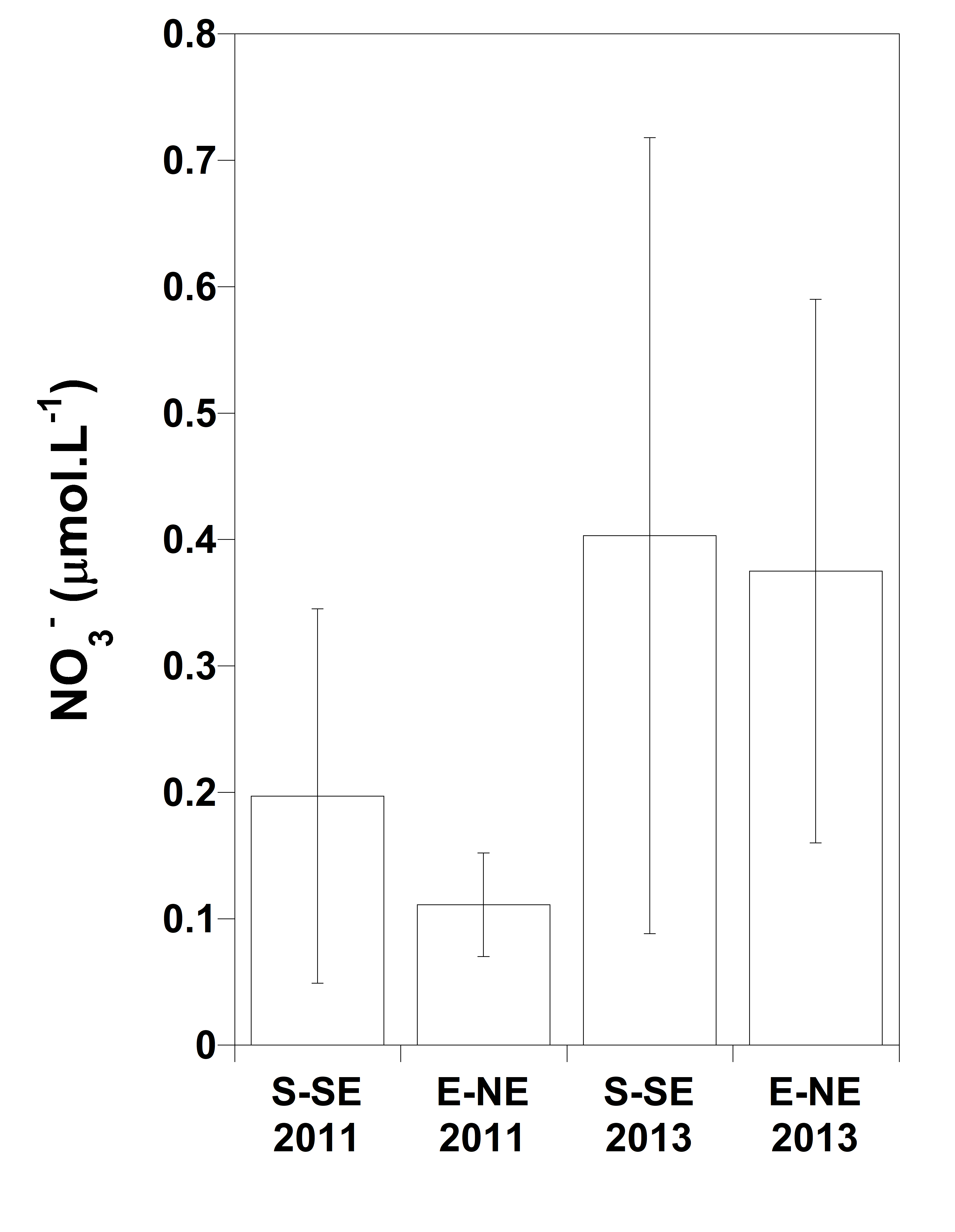

Supplement: Supplemental Information 1 — Variations in inorganic nitrate (NO3−) in surface water along the S-SE and E-NE side of the Saba Bank in 2011 and 2013 with standard deviations. [file peerj-06-5460-s001.png]

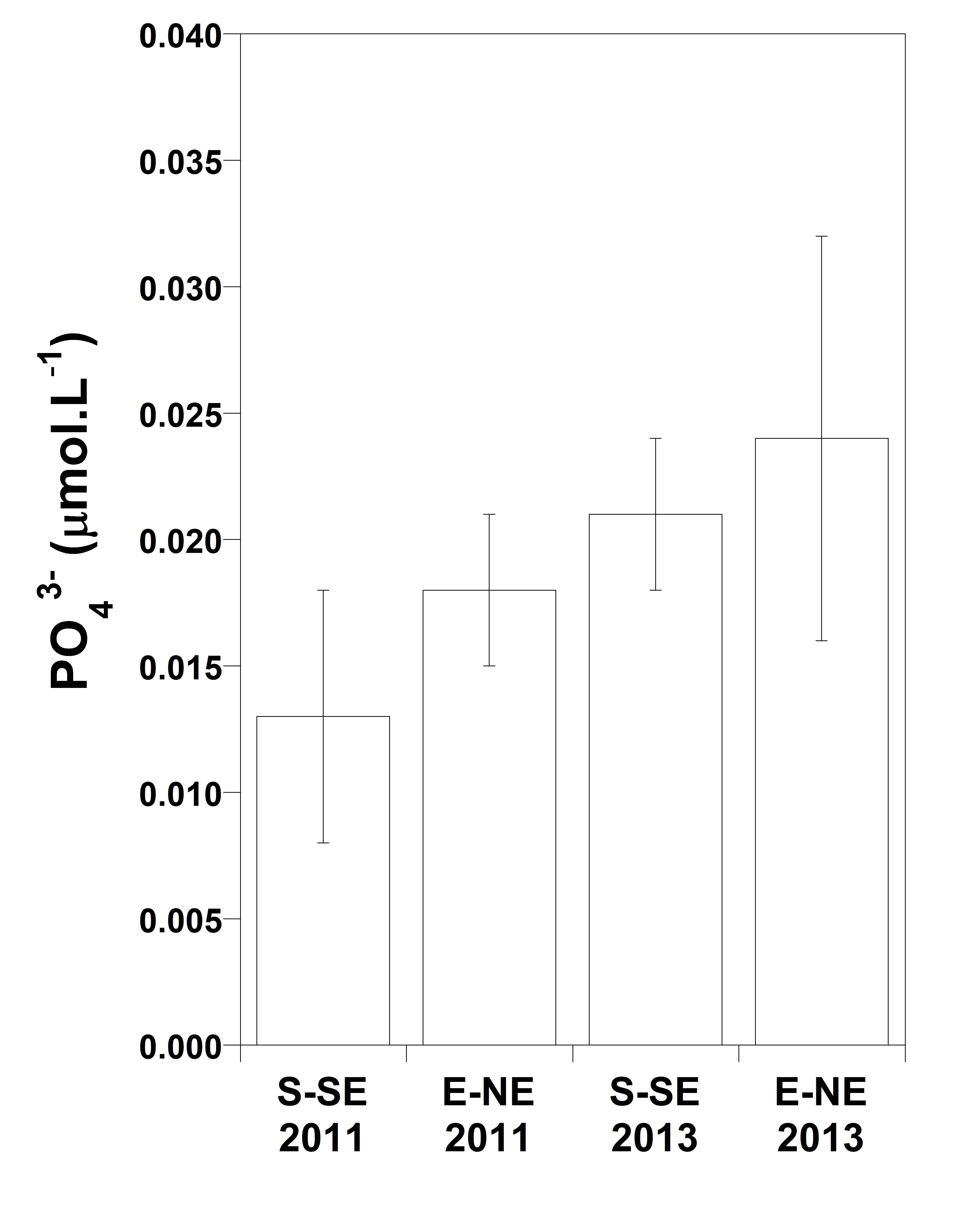

Supplement: Supplemental Information 2 — Variations in soluble reactive phosphate (PO43−) in surface water along the S-SE and E-NE side of the Saba Bank in 2011 and 2013 with standard deviations. [file peerj-06-5460-s002.png]

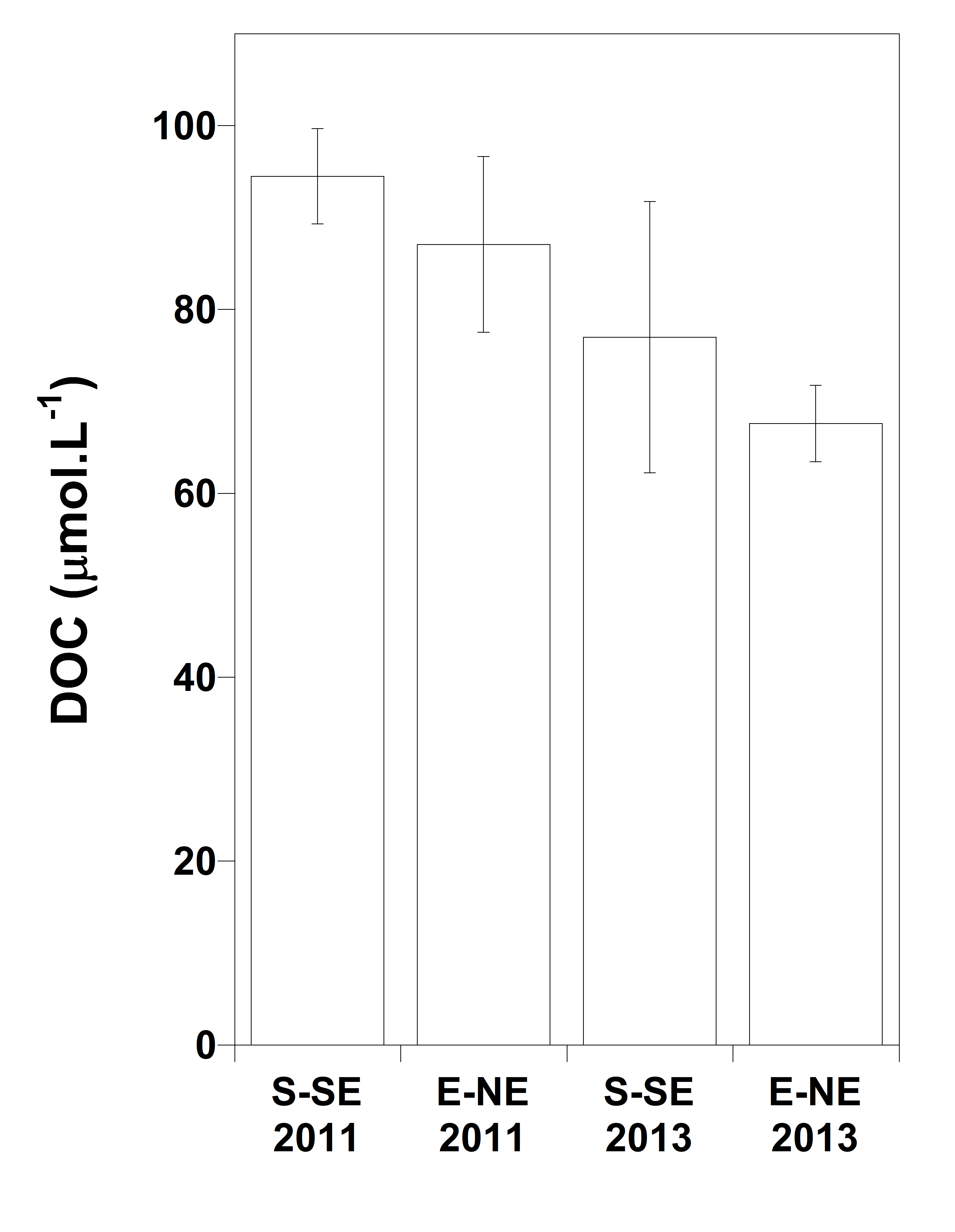

Supplement: Supplemental Information 3 — Variations in dissolved organic carbon (DOC) in surface water along the S-SE and E-NE side of the Saba Bank in 2011 and 2013 with standard deviations. [file peerj-06-5460-s003.png]

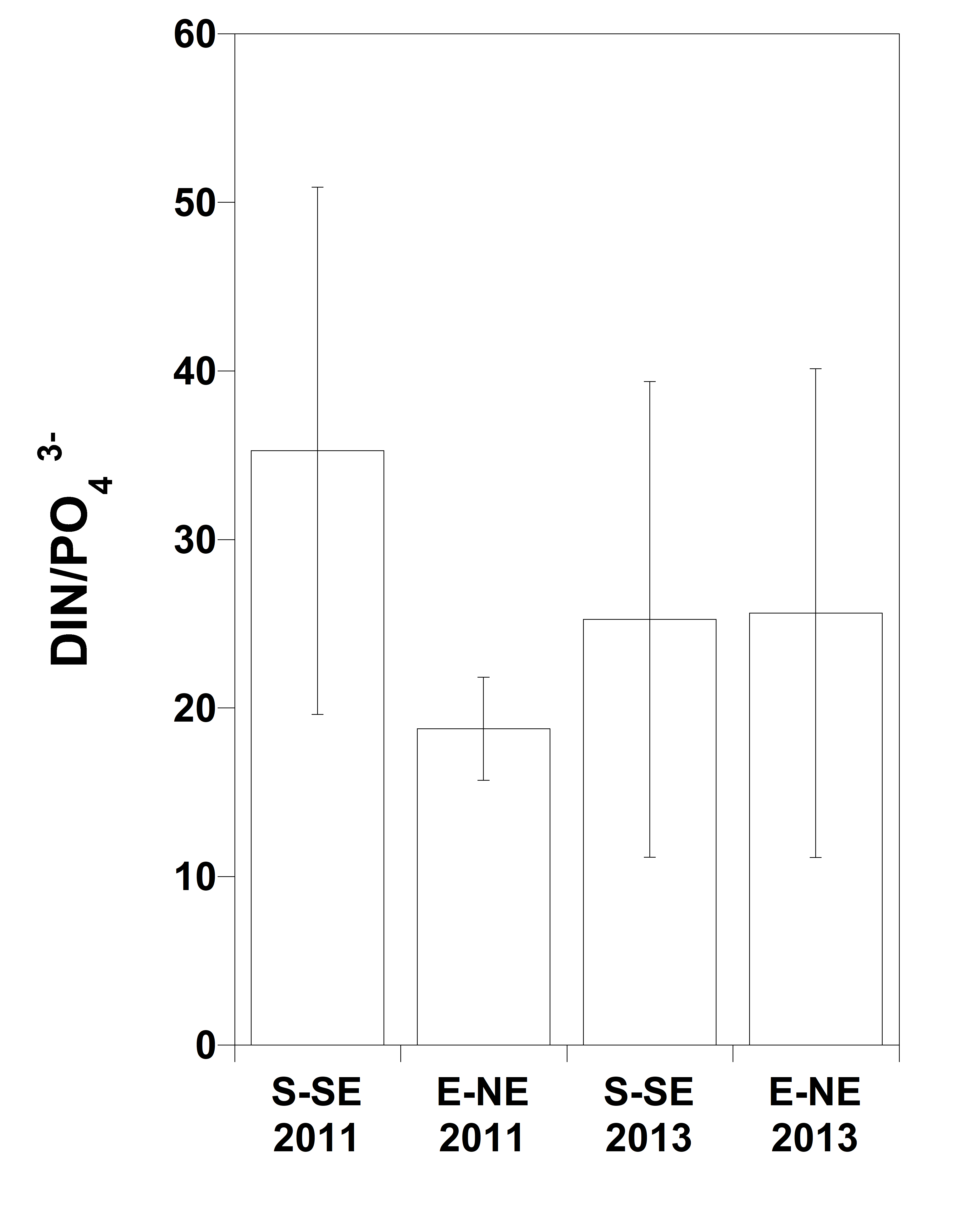

Supplement: Supplemental Information 4 [file peerj-06-5460-s004.png]
